# *Salmonella enterica* ser Saintpaul Colitis Mimicking Crohn's Disease

**DOI:** 10.1155/crgm/1627615

**Published:** 2025-07-26

**Authors:** Benjamin Gow-Lee, Phillip Bennett, Jessicia Schmitt, Amir Kashani

**Affiliations:** ^1^Division of Gastroenterology, Hepatology, and Nutrition, Department of Internal Medicine, University of Utah School of Medicine, Salt Lake City, Utah, USA; ^2^Department of Pathology, University of Utah School of Medicine, Salt Lake City, Utah, USA

**Keywords:** Crohn's disease, infectious colitis, mimics, S. Saintpaul

## Abstract

Diagnosing inflammatory bowel disease (IBD) can be challenging in the presence of mimicking conditions, such as infectious colitis. An accurate diagnosis is important to avoid unnecessary treatment. Nontyphoidal *Salmonella* species are an important cause of infectious colitis that at times can mimic IBD. *Salmonella enterica* serotype Saintpaul has caused numerous fatal foodborne gastroenteritis outbreaks worldwide. It has also been known to cause other infections, such as bacteremia, splenic abscesses, and meningitis, but has only rarely been known to cause colitis. Worryingly, antibiotic resistance rates of S. Saintpaul are rising. This case report presents a woman with S. Saintpaul colitis mimicking Crohn's disease. Despite an initial plan to start biologic therapy, long-term follow-up while off immunosuppressant therapy confirmed S. Saintpaul as the underlying cause of colitis, sparing our patient long-term immunosuppression. This case highlights the importance of ruling out infectious colitis before beginning long-term immunosuppressive therapy and the challenges of mimicking conditions as well as the novelty of the Saintpaul serotype causing colitis.

## 1. Introduction

The incidence of inflammatory bowel disease (IBD) is increasing worldwide. Diagnosing IBD can be made difficult by the presence of mimickers, such as infectious colitis. *Salmonella* species are important causes of infectious colitis. *Salmonella* are gram-negative bacilli that are clinically divided into typhoidal serovars (including *Salmonella enterica* ser typhi and paratyphi), which cause typhoid fever, and nontyphoidal *Salmonella enterica* serotypes (NTS), which usually cause a self-limited gastroenteritis in immunocompetent hosts [1]. If presenting with colitis, NTS often presents similar to other causes of dysentery (e.g., *Shigella* and *Campylobacter*) with acute, often bloody diarrhea, fever, and abdominal pain. NTS infectious colitis and IBD have a convoluted connection: NTS infectious colitis can both increase the risk of contracting IBD [2] and mimic IBD [3–5]. This mimicry has been seen with multiple *Salmonella* serotypes, including Enteritidis [6], Veneziana [7], and Java [8]. Another *Salmonella* serotype, ser Saintpaul, can also mimic IBD. This case presents a woman who was initially diagnosed with Crohn's disease but was then found to have *Salmonella* ser Saintpaul colitis. She had endoscopic and histologic disease resolution after antimicrobial therapy. She remained disease-free on long-term follow-up without further treatment.

## 2. Case Report

**Figure 1:**
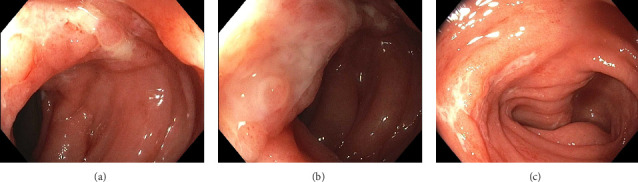
Initial colonoscopy showed ulceration and edema in the transverse colon (a) and in the descending colon (b and c).

**Figure 2:**
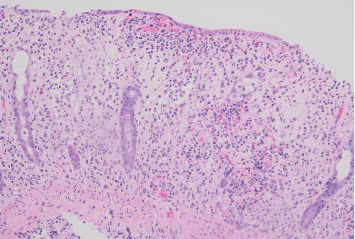
Hematoxylin and eosin (H&E) stain histology from sigmoid colon biopsies showed severe active colitis with abundant neutrophilic inflammation, along with cryptitis, crypt abscesses, and surface erosion, with the lack of significant chronic changes (H&E stain x200).

A 22-year-old previously healthy woman presented with six weeks of abdominal pain and bloody diarrhea, along with nocturnal stooling, tenesmus, and subjective fevers. She had no personal or family history of IBD. She had no recent travel, antibiotic use, or sick contacts. Upon presentation, she was afebrile but hypotensive with a diffusely tender abdomen. Laboratory findings included a normal white blood cell count, mild microcytic anemia, elevated C-reactive protein (CRP) at 8.3 mg/dL (normal: 0.0–0.8 mg/dL), and elevated fecal calprotectin at > 1250 μg/g (normal ≤ 49 μg/g). A colonoscopy revealed patchy erythematous and edematous mucosa throughout the colon and distal terminal ileum. There were numerous serpiginous ulcers from the mid-transverse colon distally to the descending colon (Figure 1). The ileocecal valve and the distal rectum each contained a solitary ulcer. Histologic findings included moderate to severe active colitis, lacking features of chronicity (Figure 2). Initial infectious stool studies were negative, including a gastrointestinal viral panel by polymerase chain reaction, *Clostridioides difficile* glutamate dehydrogenase antigen and toxin, Shiga toxin-producing *Escherichia coli*, stool ova and parasite, and stool wet mount and stain. However, stool culture grew S. Saintpaul.

Based on extended symptom duration and characteristic endoscopic findings, Crohn's disease was deemed the likely etiology over ulcerative colitis given the patchy colonic inflammation. The lack of chronic inflammatory features on histology was attributed to an evolving disease still early in the course. Infectious disease was consulted, and the S. Saintpaul, given its less invasive nature, was thought to be a superimposed infection and not the main cause of her clinical presentation. Concurrent S. Saintpaul treatment was recommended. Due to financial concerns, the patient requested to be discharged early in her course. She was discharged with a 2-week course of prednisone taper and a simultaneous 2-week course of ciprofloxacin 500 mg twice daily to treat what was thought to be the bystander S. Saintpaul infection with infectious disease guidance.

**Figure 3:**
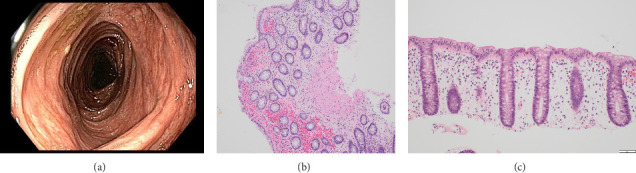
Repeat colonoscopy after antimicrobial therapy showed complete endoscopic resolution (a). Hematoxylin and eosin (H&E) stain histology additionally showed disease resolution as evidenced by a decrease in inflammatory components, lack of crypt abscesses, and no Paneth cell metaplasia or architectural distortion. (b) and (c) H&E stain x100 and x200, respectively.

On follow-up 2 weeks later, her symptoms had improved greatly. Repeat CRP and fecal calprotectin had normalized. Plans were made to start vedolizumab for steroid-sparing Crohn's treatment. However, her sustained clinical remission and complete normalization of inflammatory markers with a limited prednisone course as the sole immunosuppressant raised suspicions against a diagnosis of Crohn's disease. Therefore, it was decided to further reassess the disease activity before starting vedolizumab. A repeat fecal calprotectin six months later was < 16 μg/g (normal ≤ 49 μg/g). Unfortunately, repeat infectious studies (including viral PCR panel and stool studies) were not collected. She underwent another colonoscopy, which was endoscopically and histologically normal other than nodular terminal ileum mucosa (Figure 3). Two years after her initial presentation, she has had no recurrence of symptoms and has experienced no treatment other than her initial prednisone and ciprofloxacin.

## 3. Discussion

Our patient's 6-week history of bloody diarrhea with patchy inflammation and ulceration throughout the colon along with elevated inflammatory markers initially resulted in a diagnosis of Crohn's disease. Her eventual return of stool culture growing S. Saintpaul and complete disease resolution after a course of ciprofloxacin and short prednisone course with no continued immunosuppressive therapy support the diagnosis of S. Saintpaul colitis mimicking Crohn's disease. Unfortunately, posttreatment infectious studies were not collected to provide proof of cure. Her endoscopic and histologic disease resolution is unlikely to be from a limited course of corticosteroid therapy, as corticosteroids do not necessarily result in endoscopic remission of IBD and are more associated with a transient clinical response [9–12]. Her complete disease resolution symptomatically, biochemically, endoscopically, and histologically argues against Crohn's disease and instead supports a diagnosis of S. Saintpaul infectious colitis causing her presentation with the organism not acting as the bystander infection as it was originally thought to be but as the source for her disease.

*Salmonella* species have usually been associated with self-limited, foodborne gastroenteritis outbreaks. S. Saintpaul gastroenteritis outbreaks caused by contaminated food or water account for up to 4% of *Salmonella* gastroenteritis cases [13]. S. Saintpaul has caused outbreaks in multiple countries, including a 2008 outbreak in the United States affecting nearly 1500 people [14]. S. Saintpaul has been found in produce [15], fruit, water, and assorted meats [13] and survives well in fresh water [16]. Concerningly, various labs have found strains of S. Saintpaul that are multidrug resistant [17–19], possibly due to the discovery that S. Saintpaul has been found to contain integrons which can confer drug resistance [20]. S. Saintpaul's multiple lines of drug resistance as well as its unique profile of multiple virulence genes help make it distinct from other serotypes of *Salmonella enterica* [19, 21]. The incidence of S. Saintpaul is higher outside the United States, but given that high amounts of imported birds were found to have S. Saintpaul [22], it is reasonable to assume that the incidence of S. Saintpaul will continue to rise domestically. Besides gastroenteritis, S. Saintpaul has also been known to cause bacteremia [23], splenic abscesses [24], meningitis [25], and spondylitis [26] in humans. In addition, S. Saintpaul was once reported to have caused a rapid death from bacteremia with an additional finding of a nonulcerating colitis on autopsy [27]. This nonulcerating colitis was the only other case of S. Saintpaul colitis published in the literature. To our knowledge, our case is the first reported case of S. Saintpaul causing an ulcerating colitis and mimicking IBD.

Clinically, NTS tends to cause self-limited gastroenteritis in immunocompetent individuals that seldomly requires antimicrobial treatment [1, 11]. It is the most common bacterial cause of diarrhea worldwide [11]. Patients develop symptoms usually within 48 h of NTS exposure [12]. Common symptoms include nausea, vomiting, and diarrhea ranging from loose stools to bloody stools. If limited to gastroenteritis, symptoms usually last for 3–5 days but can last for 2–3 weeks in patients with enterocolitis [12]. Less than 1% of infected patients become chronic carriers. This risk is increased if patients have structural biliary or urinary tract abnormalities [28]. Risk factors for developing NTS diarrhea include immunosuppression, being at the extremes of age, past antibiotic use, and reduced gastric acid secretion (whether through medication, achlorhydria, etc) [29]. NTS species can cause bacteremia in 5% of patients, especially in those with the above risk factors [5]. Interestingly, patients with NTS diarrhea are at increased risk of contracting IBD [2, 30].

Treatment of NTS infections is case dependent. Multiple studies have found no benefit in treating gastroenteritis caused by NTS [31, 32]. Unnecessary treatment of gastroenteritis increases the risk of antibiotic resistance and adverse effects and may even extend how long the bacteria are shed in feces. However, gastroenteritis treatment is recommended in severe cases and in immunocompromised hosts [32]. In contrast to *Salmonella* gastroenteritis, treatment for *Salmonella* colitis is recommended [33]. In the United States and Europe, NTS retain susceptibility to fluoroquinolones [5].

This case presents an important treatment consideration for IBD. Based on our patient's presumed good response to anti-inflammatory treatment with corticosteroids, she was almost subjected to long-term biologic treatment. Fortunately, reassuring new blood and stool inflammatory studies prompted a repeat colonoscopy, which confirmed disease resolution, sparing her from biologic treatment. Should our patient have started vedolizumab treatment for Crohn's disease as was previously planned, subsequent normal colonoscopy would be assumed to represent a good response to biologic therapy. This could have subjected the patient to extended treatment with immunosuppressants with all the risks they bring.

In addition, treating an infectious colitis mimicking IBD with immunosuppressive drugs can have disastrous, sometimes fatal, consequences, especially if immunosuppressive medications are used as monotherapy [34]. In our patient's case, she was treated on discharge with both ciprofloxacin and immunosuppressing prednisone. This was due to both our and the infectious disease service's assessment of Crohn's disease as the primary driver of our patient's presentation and the S. Saintpaul as a mere superimposed infection. If our initial diagnosis of Crohn's disease had ultimately been her true diagnosis, this dual treatment of her underlying inflammatory disorder and superimposed infection would have been reasonable. When infectious colitis is tentatively diagnosed in a patient with other findings suspicious for IBD, close follow-up to assess response to antimicrobial therapy can clarify the diagnostic picture. This is the approach we pursued, which ultimately supported her final diagnosis of infectious colitis. Persistent gut inflammation, symptoms, and biochemical, endoscopic, and histologic evidence of disease, despite appropriate therapy and microbe eradication, favor a diagnosis of IBD. A summary of typical presentation differences between Crohn's disease and *Salmonella* colitis has been included in Table 1.

S. Saintpaul, like many other types of infectious colitis, may mimic IBD, hindering accurate diagnosis and treatment. In such cases, it is prudent to consider patients' symptoms in response to antimicrobial therapy to guide further laboratory or endoscopic workup to confirm the diagnosis of IBD and treat patients appropriately. This case additionally highlights the Saintpaul serotype causing infectious colitis, a rare presentation.

## Figures and Tables

**Table 1:** Differences in typical presentation of Crohn's disease and *Salmonella* colitis [35–38].

	Crohn's disease	*Salmonella* colitis
Clinical presentation	Chronic diarrhea Abdominal pain Fatigue	Acute bloody diarrhea Fever Abdominal pain Often associated with outbreaks

Endoscopic presentation	Discontinuous involvement/skip lesions Rectal sparing Deep, linear, serpiginous ulcers Strictures Fistulas	Fine ulcerations and erosions Possible rectal involvement

Histologic features	Transmural inflammation Granuloma formation Crypt distortion	Acute inflammation Crypt architecture preservation

## Data Availability

The clinical data used to support the findings of this study are included within the article.
